# Extended Producer Responsibility and Product Stewardship for Tobacco Product Waste

**DOI:** 10.4172/2252-5211.1000157

**Published:** 2014-09-04

**Authors:** Clifton Curtis, Susan Collins, Shea Cunningham, Paula Stigler, Thomas E Novotny

**Affiliations:** 1 Director, The Varda Group; and Policy Director, Cigarette Butt Pollution Project, USA; 2 President, Container Recycling Institute, USA; 3 Sustainability Policy, Research & Planning Consultant, Container Recycling Institute, USA; 4 Assistant Professor, University of Texas Health Sciences, San Antonio Regional Campus, USA; 5 Chief Executive Officer, Cigarette Butt Pollution Project and Professor of Epidemiology, Graduate School of Public Health, San Diego State University, USA

**Keywords:** Tobacco control, Tobacco product waste, Cigarette butts, Producer responsibility, Product stewardship

## Abstract

This paper reviews several environmental principles, including Extended Producer Responsibility (EPR), Product Stewardship (PS), the Polluter Pays Principle (PPP), and the Precautionary Principle, as they may apply to tobacco product waste (TPW). The review addresses specific criteria that apply in deciding whether a particular toxic product should adhere to these principles; presents three case studies of similar approaches to other toxic and/or environmentally harmful products; and describes 10 possible interventions or policy actions that may help prevent, reduce, and mitigate the effects of TPW. EPR promotes total lifecycle environmental improvements, placing economic, physical, and informational responsibilities onto the tobacco industry, while PS complements EPR, but with responsibility shared by all parties involved in the tobacco product lifecycle. Both principles focus on toxic source reduction, post-consumer take-back, and final disposal of consumer products. These principles when applied to TPW have the potential to substantially decrease the environmental and public health harms of cigarette butts and other TPW throughout the world. TPW is the most commonly littered item picked up during environmental, urban, and coastal cleanups globally.

## Introduction

The human health effects of smoking are well known, but far less is known about the environmental impacts of tobacco product waste (TPW), especially cigarette butts. This paper addresses the environmental concerns regarding TPW throughout its lifecycle, with special emphasis on cigarette butt waste. The lifecycle environmental issues for tobacco include the growing process (with concerns for heavy pesticide and petroleum-based fertilizer use, land degradation, and deforestation) [1,2], as well as production (manufacturing, packaging, and distribution wastes)[3]; and consumer use (including CO2 production, methane release, second hand smoke exposure, and third-hand smoke effects[4]), and finally, disposal of cigarette butts and packaging as TPW[5,6].

There were an estimated 5.5 trillion cigarettes sold globally in 2011, with approximately 293 billion sold in the United States [7,8]. By some estimates, at least one-third of all cigarettes smoked are tossed into the environment, comprising by far the largest single type of litter by count, about 30–40% of all items picked up, in coastal and urban cleanups dating back to the 1980s[9].

Beginning in the 1950s, the tobacco industry shifted production of manufactured cigarettes from unfiltered to filtered, using a variety of different components. The filtered cigarettes were marketed as being “healthier” in response to the new concerns for the health risks of smoking [10]. Since at least the 1990s, over 98% of all cigarettes sold in the United States are filtered, and nearly all of the filters sold are made of cellulose acetate, a separately manufactured plastic element that is attached to the tobacco product [11,12]. The increase in production and the fraudulent marketing of filtered cigarettes as a healthier option for smokers over the last 60 years presents us with not only a public health problem due to the filter fraud, but also an environmental concern with the non-biodegradable filters that are the primary component of discarded cigarette butts.

The US National Cancer Institute reviewed the changing cigarette product, in particular ‘light’ and ‘low-tar’ designations, and concluded that “Epidemiological and other scientific evidence, including patterns of mortality from smoking-caused diseases, does not indicate a benefit to public health from changes in cigarette design and manufacturing over the last fifty years”[13]. This design specifically refers to the filtered cigarette, and thus discarded cigarette butts, especially the plastic filters, may be considered a dispersed source of nonbiodegradable, toxic environmental waste that could be subject to elimination without concern for the health effects of this product change [14]. Filters are still believed by many smokers and non-smokers to be health-protective devices, but there have been no benefits to public health from filters, and in fact the risks for lung cancer and chronic pulmonary disease due to smoking have actually increased since becoming widely used by uninformed smokers.

The cost to municipalities to clean up TPW is substantial. The City and County of San Francisco studied the costs of litter cleanup and disposal in 2007–2009 and estimated the costs attributed to TPW to be $22 million annually [14]. A separate study, funded by the US Environmental Protection Agency (EPA), estimated total cleanup, prevention and disposal costs of all sources of litter (including TPW) at over $500 million for West Coast communities [15]. From an environmental perspective, aquatic ecosystems, such as shorelines and waterways, may be very vulnerable to the environmental impact of TPW, as so much of this waste is deposited on land and ultimately flows downstream via storm drains, rivers, creeks and other pathways to those environments [16].

Under specific circumstances of sunlight and moisture, the filter component of cigarette butts may be broken into smaller plastic pieces that also contain and leach out some of the seven thousand chemicals contained in a cigarette [17]. Many of these chemicals, such as ethyl phenol, heavy metals, and nicotine, are in themselves environmentally toxic, and at least 50 are known human carcinogens [18]. TPW leachates have in fact been shown to be of environmental concern, with measureable amounts of heavy metals such as cadmium, arsenic, and lead in laboratory analyses [19]. They have been found to be acutely toxic to freshwater micro-organisms, with the main lethal chemicals being nicotine and ethyl phenol [20]. Recent studies using standardized EPA toxicity assessment protocols have shown that cigarette butts soaked in either fresh or salt water for 96 hours have a Lethal Concentration 50 (killing half the exposed test fish) of about one cigarette butt per liter [21].

In a May 2011 editorial in the international public health journal, Tobacco Control, tobacco control advocates and scientists urged key stakeholders “to join forces and find solutions for eliminating this especially toxic form of [cigarette butt-related] trash”[22].” The Washington, DC-based Legacy Foundation, which helped fund that special journal supplement, then convened a national webcast focusing on how public health experts, policy leaders, environmental, and community leaders can eliminate toxic TPW [23].

This paper reviews Extended Producer Responsibility (EPR), Product Stewardship (PS), and two additional related environmental principles as possible approaches to TPW prevention, reduction, and mitigation. We will also review criteria that may apply in deciding whether TPW may adhere to EPR/PS. We then present three case study summaries of EPR/PS approaches that have been used with other environmentally harmful products. Finally, we propose ten policy actions that can help prevent, reduce, and mitigate the potential environmental impacts of TPW.

## Review of Extended Producer Responsibility, Product Stewardship, and Other Related Environmental Principles

### Extended Producer Responsibility

**Figure 1:**
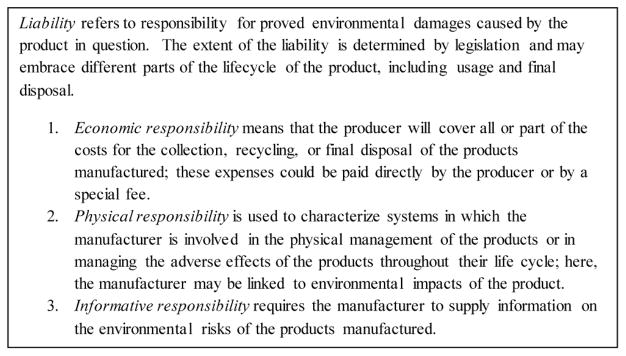
Categories of Extended Producer Responsibilities [24].

The EPR concept dates to the early 1990’s when Thomas Lindhqvist, a Swedish graduate student, prepared a report for Sweden’s Ministry of the Environment that called for making manufacturers of products responsible for the entire lifecycle of the products they produce [24]. Lindhqvist defined EPR as “an environmental policy protection strategy to reach an environmental objective of a decreased total environmental impact from a product, by making the manufacturer of the product responsible for the entire life-cycle of the product and especially for take-back, recycling and final disposal of the product.” Three central tenets embedded in this concept were:

To internalize the environmental cost of products into their retail price.
To shift the economic burden of managing toxicity and other environmental harm associated with post-consumer waste away from local governments and taxpayers and on to producers.
To provide incentives to producers to incorporate environmental considerations in the design of their products.
Lindhqvist’s focus included those three tenets, as well as four specific categories of responsibility (Figure 1).

EPR-based laws have been enacted in more than 20 US states, with legally binding features requiring manufacturers of products containing toxic or environmentally unsustainable materials to take responsibility for management throughout key parts of their lifecycle, especially for management of post-consumer waste [25]. The products addressed are diverse, including: paint, batteries, beverage containers, pesticide containers, electronics, packaging, cell phones, sharps, carpets, fluorescent lighting, mercury thermostats, radioactive devices, motor oils, mattresses, plastic bags, photographic film, smoke detectors, and auto switches.

Internationally, EPR laws and regulatory systems have been implemented in several countries, including Canada, the European Union member states, Australia, New Zealand, Japan, Sweden and Norway [26]. As with U.S. states, international approaches vary widely with respect to specific producer, consumer, retailer, and government responsibilities for end-of-life product management.

There are three reasons why producers should assume EPR for TPW management at the end of tobacco product life [27]:

Shifts waste management responsibilities and costs from local governments and taxpayers back to the polluter/producer, which in most instances is in the private sector;
Economic costs of TPW management may encourage manufacturers to design non-toxic, non-hazardous products; and
Internalizing the costs of waste product management to the producer will be fairer overall when net external costs to the public and communities are taken into account.

While all of these rationales are understandable with respect to shifting economic responsibility to producers of most consumer products, pursuing tobacco product design changes to reduce TPW toxicity are unlikely to be effective. The tobacco product is inherently hazardous to human health and contains many chemicals that are on the Agency for Toxic Substances and Disease registry priority list of hazardous substances as well as the State of California Proposition 65 list of chemicals known to cause cancer [24,28]. Chemicals covered by these lists are those that cause one or more of the following: cancer or other chronic human health effects, significant adverse acute human health effects, or significant adverse environmental effects. TPW will remain filled with these chemicals, no matter how the tobacco product or filter is altered.

### Product Stewardship

**Figure 2:**
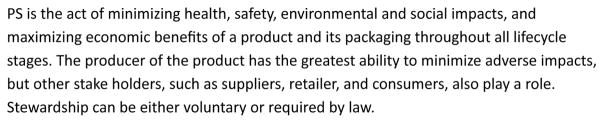
Joint Statement on Product Stewardship Principals by the Product Policy Institute, PS Institute, and California PS Council, 2012.

PS contrasts with EPR in that PS may involve other actors along the supply and retail chain, whereas EPR focuses all the responsibility for waste management onto manufacturers [29,30]. During the early-to-mid 1990s, the idea of shared responsibility, also referred to as “product responsibility,” began generating attention. PS was possibly introduced by industry as a way to dilute the EPR concept and share responsibility rather than have all responsibility fall to the producers [31]. PS is usually designed as a voluntary system that shares responsibility for the adverse environmental effects of products by all parties involved in the lifecycle [32]. PS principles therefore require much wider and more diverse involvement of parties than does an EPR-only-based approach (Figure 2) [33].

A key variable determining whether a product management system may be eligible for EPR and/or PS involves the funding system that is adopted for these approaches. With EPR, costs are to be paid by the producer; when the program is a cost-sharing arrangement between producers and other stakeholders, it would be thought of as a PS approach. To date, the tobacco industry has denied any form of producer responsibility for TPW, shifting almost the entire focus onto the consumer. For example, in formerly secret tobacco industry documents, Philip Morris companies, Inc. (1998) described their position on EPR as follows:

“The Company opposes the concept of manufacturer/producer responsibility when defined to mean that the manufacturer/producer must accept sole and complete responsibility for a product/package throughout its life cycle. Specifically, it’s the Company’s position that these waste management practices….create a highly inefficient system due to the fact that responsibility continues even after the manufacturer has relinquished control of the product and/or package. Consistent with our Environmental Principles, we commit to provide consumers with appropriate and useful information on the environment and their role, as well as that of communities and business, in becoming part of environmental solutions, including those which affect solid waste management” [34].”

### Other Environmental Principles that May be Applicable to TPW

The Polluter Pays Principle (PPP) was introduced in the 1970s by the Organization for Economic Cooperation and Development (OECD) in consideration of the economic costs associated with protection of the environment. As framed, the PPP “[meant] that the polluter should bear the expenses of carrying out [pollution prevention and control] measures decided by public authorities to ensure that the environment is in an acceptable state [35].” A 1989 OECD initiative, dealing with accidental pollution, made specific reference to “hazardous” components when invoking the use of PPP [36]. The PPP integrates environmental protection, social development, and economic activities by using market and/or regulatory instruments to ensure that persons or organizations responsible for pollution bear the full environmental and social costs of their activities, and that those costs are reflected in the market price for goods and services. Over time, the PPP has become a generally accepted principle of international environmental law and policy, perhaps most advanced in its application within the EU, in focusing attention and responsibility on polluting sources. In line with resistance to EPR, there is no evidence that PPP has applied to tobacco industry responsibility for TPW.

The Precautionary Principle is based on the caution that governs many aspects of daily life, and responds to the complexity of environmental risks to health and the often indeterminate nature of cause-and-effect relationships between potentially hazardous waste products and health effects. This principle first appeared in the 1970s as a basis for water protection policies in Germany. Over the years it has provided an overarching framework for addressing threats from toxic chemicals involving a wide range of exposures [37]. At its core, this principle calls for preventive, anticipatory measures to be taken when an activity raises threats of harm to the environment, wildlife, or human health, even if cause-and-effect relationships are not fully established. The principle is instructive with regard to TPW, given the evidence that this waste stream has toxic, carcinogenic, and otherwise harmful chemicals derived from tobacco products and the attached cellulose acetate filters. As such, prevention, reduction and mitigation efforts involving TPW could be undertaken to help prevent potential TPW-related harm to humans, animals, and ecosystems before it is evident. Moreover, application of this principle would include not only current TPW prevention and reduction, but would apply to past polluting practices that have produced environmentally persistent TPW such as cigarette filters and plastic packaging.

## Review of Criteria for Applying EPR/PS

**Figure 3:**
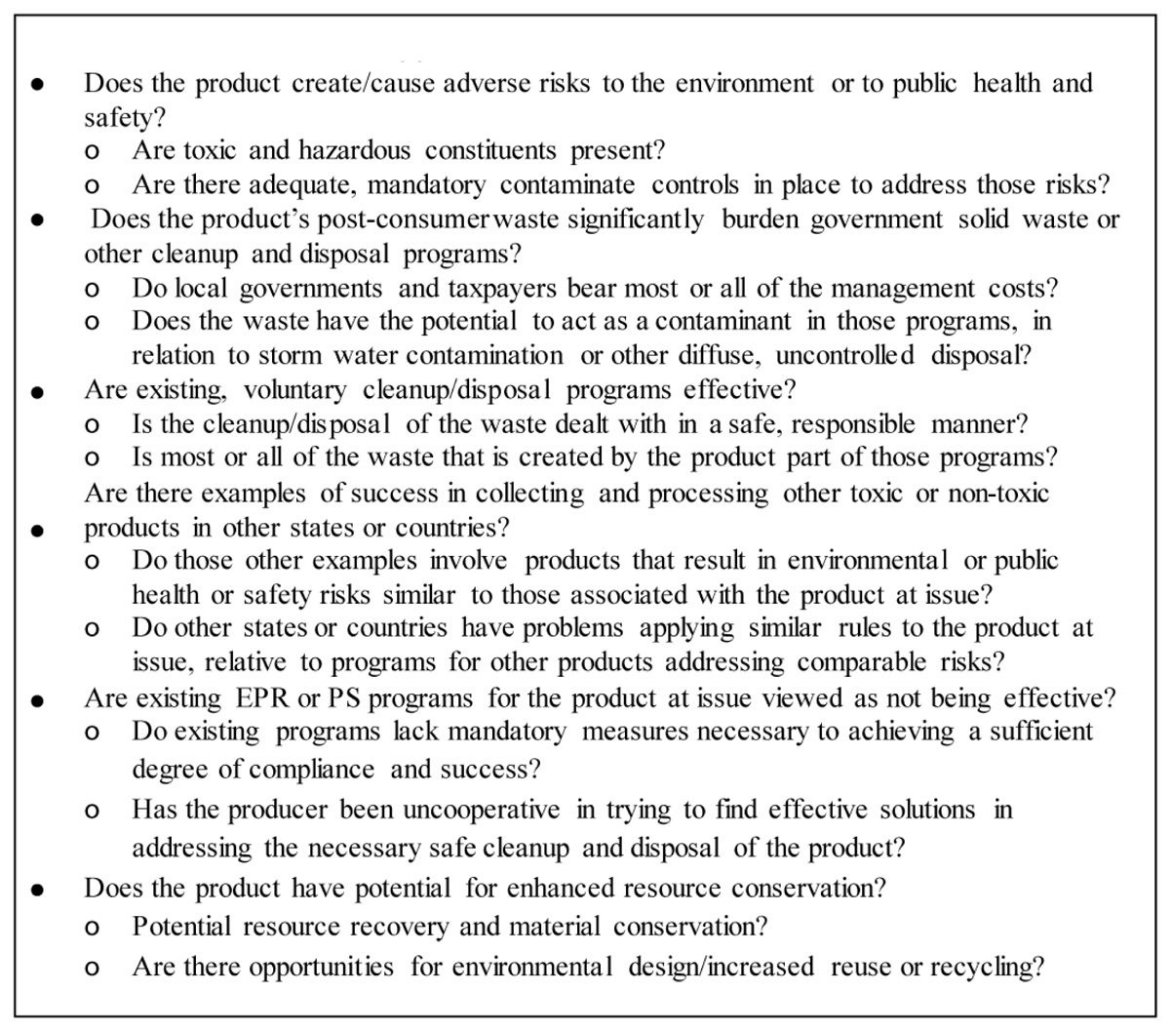
Criteria for EPR/PS Approaches to Consumer Product Waste

A variety of criteria may be applicable in determining whether TPW should adhere to EPR and complementary PS principles and standards. The six criteria mentioned below are framed as questions for which the answers provide a sense of whether any consumer product waste should qualify for an EPR/PS based policy, legal, regulatory, or voluntary regime (Figure 3) [38,39].

Criteria similar to these have been used in several states, including California, Oregon, and Washington, to determine whether consumer product wastes should be managed through EPR and/or PS approaches [40,41,42]. With regard to TPW, the end-of-life tobacco product phase is a strong candidate for use of EPR/PS. As noted earlier in relation to the core tenets of EPR framed by Lindhvqist, TPW will not be amenable to resource recovery/conservation or environmental design as described in the final criterion in Figure 3. Although detoxification, biodegradation, and disposal strategies may be among the best options for EPR/PS approaches to many other consumer products, TPW may need more novel approaches, given its ubiquity and the specific toxic, harmful environmental contaminants it produces.

## Relevant Case Studies

EPR/PS have been applied to a variety of products, including beverage containers, paint, and batteries. Many other products are also candidates for application of those environmental principles, especially for products with similar toxic characteristics. We present three case study summaries that may inform EPR/PS-related strategies for TPW (full case studies are available from the authors).

### The Oregon PaintCare Stewardship Program

Leftover paint is the largest component of household hazardous waste (HHW) in the United States. The EPA estimates that about 10% of all paint purchased each year (approximately 64 million gallons) goes unused [43] According to the EPA, municipal governments, which bear the managerial burden of leftover paint collection, could avoid more than a half billion dollars annually in mitigation costs with a paint stewardship program managed by the paint industry and funded by consumers.

Oregon enacted the first paint stewardship law in the United States in 2009 and followed up with strengthening amendments in 2013 [44]. Its PaintCare program requires paint manufacturers to implement a cost-effective and environmentally sound program for managing leftover paint. The program mandates a recycling fee at the point of sale for paint in five-gallon or less containers; these fees must be sufficient to cover all program administrative and recycling costs. At the end of the first two years of the program, collection sites numbered more than 100, most of which are located in retail paint stores. During that same time, the quantity of paint collected and reprocessed increased by 34%. Oregon’s PaintCare program uses a solid-waste management hierarchy similar to that followed by the EPA, which focuses sequentially on source reduction, reuse, recycling, recovery, and biodegradation [45].

While the Paintcare program overall has received high approval ratings, several challenges required amendments to the program; the program in Oregon was made permanent in June 2013. PaintCare programs have now been established in California and Connecticut, and four other states have passed similar legislation (Rhode Island, Vermont, Minnesota and Maine).

Stewardship-related elements of PaintCare in four areas are very relevant and worthy of replication in addressing TPW issues:

#### Creation of a stewardship organization

Similar to features in Oregon’s state law addressing leftover paint, a TPW stewardship entity could be established at the state level, as a corporation or nonprofit, created by the tobacco industry. This would be accorded the legal mandate and responsibility to implement an industry-sponsored program, with oversight provided by the state’s environmental quality/protection department or agency. Given the health-related issues involved with TPW, consultation with a state’s health department or agency would be recommended.

#### Access to convenient collection sites

Establishment of Hazardous Household Waste and retail collection sites for TPW. Given the toxic, poisonous nature of the substantial quantities of filters and remnant tobacco and paper discarded randomly, though, TPW protective recovery paraphernalia would likely be needed in gathering and returning TPW to collection sites.

#### Educational and outreach activities

A TPW stewardship organization could promote activities, including but not limited to signage, written materials and templates for reproduction, shared with retailers for distribution to the consumer at time of sale. Also, they could identify collection opportunities, and promote waste management hierarchy, especially technologies and management of biodegradation and safe, secure disposal, consistent with other applicable laws.

Plans, annual reports and program/budgeting: Documentation tasks involving required review and approval by an oversight department should be part and parcel of the responsibilities associated with any TPW program. Having in place supervisory review, guidance and sign-off, independent yet deeply familiar with and interested in the operations of the program, would help significantly to facilitate and ensure its accountability and success.

### British Columbia Beverage Container Recycling Program

The Litter Act of 1970 in British Columbia was the first beverage container deposit law, and the first EPR law in North America; the law is now called the Recycling Regulation, and litter concerns were a primary reason for passage of this law [46]. This container recycling program now requires a mandatory deposit on every beverage container offered for sale (with minor exceptions). Consumers pay the deposit at the time of purchase, and the deposit amount appears on their receipt. Consumers return empty beverage containers to retail stores or special take-back locations (“depots”), and they receive the full amount of the deposit in return. This program achieves a recycling rate of 80% or more.

Such a container deposit law places a monetary value on beverage containers, and this value then reduces litter in two ways: (1) people are less likely to litter, because the container can be returned for a refund, and (2) if they do litter, another person is likely to pick up the container and return it to receive the refund. Data from the Great Canadian Shoreline Cleanup indicate that beverage container litter is about 30% lower in British Columbia than in the provinces of Manitoba and Ontario, where there are no mandatory container deposit-refund laws [47].

Recycling centers, where consumers return beverage containers to the depots for recycling and refilling, are licensed for a specific geographic area. Consumers may also return limited numbers of containers to grocery stores and liquor stores. The beverage producers operate the deposit-refund system in British Columbia, and there are no statutory fees or charges remitted to government under the system. To carry out deposit-refund obligations within a province-wide system, beverage producers formed two stewardship organizations to manage program operations. These stewardship organizations are answerable to the Provincial Ministry of Environment, the agency authorized to carry out the Recycling Regulation. This Ministry approves the stewardship organization program plans as well as annual reports and five-year updates.

Among the 20 states with EPR-based laws, the beverage container deposit laws adopted first in Oregon and Vermont in the 1970s to reduce container litter are noteworthy. Prior to this, the vast majority of packaged beverages were sold in refillable bottles, and consumers returned those containers to retrieve deposits. In the 1960s, the ownership and distribution streams for beverage companies were consolidated, and these companies almost completely embraced single-use containers. There are now beverage container deposit laws in 10 U.S. states and the Territory of Guam, in 10 Canadian Provinces, and in more than 20 other countries worldwide.

A deposit-return scheme for TPW may only be feasible if the toxicity of the returned TPW can be managed. TPW differs significantly from beverage containers because of esthetics (odor), toxicity of the chemicals exuded from TPW, and the special care that may be necessary with regard to disposal and transport of this toxic waste material. Thus, other models of product stewardship for toxic and/or hazardous waste products may be more applicable.

## Recycling Household Batteries in Canada

Many household batteries are classified as hazardous waste and contain a number of heavy metals and toxic chemicals such as mercury, lead, and cadmium. When these batteries are incinerated or deposited in landfills, they can contaminate soil and waterways, and they may present a risk to human health [48]. Battery recycling aims to reduce the number of batteries disposed of as municipal solid waste.

Canada’s legislation and management of used batteries is conducted on a province-by-province basis. However, the responsible parties for collecting and recycling used batteries are the manufacturers, brand owners, or first importers. These businesses joined collectives and established two non-profit stewardship organizations: US-based Call2Recycle (operating in all provinces) and Stewardship Ontario (Ontario only) [49]. These organizations are financed by manufacturers on a market share-based-reimbursement arrangement. Neither organization receives funding from the government. There are now approximately 7,000 used battery drop-off sites at retail centers, public agencies, community centers, and businesses in British Columbia, Manitoba, Ontario, and Quebec.

Ontario, in fact, has an extensive list of HHW’s that must be recycled or taken back. These include: antifreeze, lubricating oil and filters, fertilizers, paints, solvents, and single-use dry batteries. The recycling drop-off service (known as Orange Drop) is free to consumers (www.makethedrop.ca). In addition to the drop off service, Stewardship Ontario funds that province’s Battery Incentive Program (BIP), which pays transporters for returning recycled batteries. Ontario’s battery take-back system has resulted in battery recycling rates of 12%, and these are the highest in North America [50].

Call2Recycle also recycles rechargeable batteries in the province [51]. Contractors receive the used batteries at their warehouses, record details about the weight and battery types of the shipment, and then separate the batteries by chemical content. The batteries are then shipped to the appropriate specialty processors by chemical type. The processors extract usable chemicals and metals to be used in the manufacture of new products. Waste products are disposed of according to Responsible Recycling and Basel Action Network standards [52].

Like spent batteries, TPW contains toxic components (ethyl phenol, nicotine, heavy metals, and many carcinogens) that can have detrimental effects to human health and the environment. Thus, a take-back program, as demonstrated in Canada for spent batteries, may be applied to TPW.

## EPR/PS-Related Policy Actions to Prevent, Reduce, and Mitigate TPW

To expand on these findings, we next present ten policy approaches based on the weight of evidence regarding the environmental impact of TPW thus far.

### Extended producer responsibility and product stewardship laws and programs

TPW prevention, reduction, and mitigation could be made the responsibility of the tobacco industry as well as other parties in the lifecycle of tobacco product sales and usage through EPR/PS. These could be legally binding and/or voluntary programs for cleanup, take-back, and final disposal. In addition, public agencies tasked to regulate the stewardship agencies, similar to those involved in the battery recycling case study, must ensure follow-through on obligations by benchmarking, setting financial and operational reporting standards, requiring transparency, requiring annual reporting and third party audits, and establishing mechanisms for public input and continual improvement.

### Bans of single use, disposable filters

Sales of some products known to be hazardous or prone to improper disposal have simply been banned by state-level authorities (e.g., pop-tops on aluminum cans, plastic tampon applicators, etc.). Given that cellulose acetate cigarette filters are not biodegradable and may cause significant environmental degradation, and given their toxicity, persistence, and ubiquity, a sales ban on single-use filters on cigarettes would reduce a significant portion of TPW. Unfortunately, tobacco remnants from unfiltered cigarette butt waste will still leach out some toxicants, but the removal of the plastic filter will reduce a significant volume of TPW while reducing the time needed for any biodegradability of the tobacco remnant.

### Bans on outdoor public smoking

Laws that ban smoking vary widely across the United States, with some states banning it in certain areas and others banning it nearly everywhere. According to a 2013 report of the American Nonsmokers’ Rights Foundation [53], more than 80% of the U.S. population now lives under a ban on smoking in “workplaces, restaurants, and/or bars, by a state, commonwealth, or local law,” though only 48.7% live under a ban covering all workplaces, restaurants, and bars. In addition, as of April 5, 2013, at least 1,159 U.S. colleges or universities have adopted 100% smoke free campus policies [54]. Overall, these restrictions on smoking serve to change the social norm against public smoking and may also reduce the burden of TPW in outdoor environments if properly enforced. On the other hand, as smokers must move outdoors to smoke, more TPW is deposited onto streets, parks, and other public outdoor spaces, and this is then more likely to wash into storm drains and aquatic environments.

### Product Labeling

Some products carry warnings not to litter the product or packages, but this intervention has never been used to inform smokers about the non-biodegradability of filters or tobacco packaging waste. Under the 2009 Family Smoking Prevention and Control Act [55]the Food and Drug Administration could require a label of sufficient size that simply states: “Cigarette filters are non-biodegradable toxic waste. Safe disposal should be required in accordance with state law.” Additional information could also describe potential toxicity of TPW, methods for safe handling, and applicable fines for littering.

### Litigation against the Tobacco Industry

To date, most litigation against the tobacco industry has focused on health care costs [56]. Similarly, the industry could be held responsible for the environmental costs associated TPW cleanup. Litigation has been pursued against manufacturers of products that damage the environment, with those lawsuits typically based on negligence and nuisance-related legal theories involving proof of the defendant’s wrongful conduct, for failure to take reasonable steps to prevent harm, or for protecting someone’s right to use and enjoy real property. Given the accumulating evidence for the toxicity of TPW, the tobacco industry may be considered a toxic waste generator, and thus they may be liable for the costs of safe clean-up, take-back, or disposal of their products.

### Litter fees

TPW cleanup and disposal costs are substantial at local municipal levels. As noted earlier, local authorities may apply litter fees as part of a program framework to recover cleanup and abatement costs, to conduct public education, and to administer the program [13].

### Deposit/Return

Similar to beverage container deposit laws, cigarettes could be sold with a “butt deposit” to be refunded when the cigarette butts are returned to the vender. The challenge in such a program would be to develop safe transport and destruction mechanisms for TPW as part of a take back and disposal regime.

### Waste fees

Concern about toxic waste resulting from contaminated products has given rise to consumer-funded Advanced Recycling Fees (ARF) [57]. Assessed at the point of purchase, such fees can help cover the costs of recycling the item and properly disposing of non-recyclable material. ARFs differ importantly from EPR approaches in that ARFs are set by the government as a fixed fee paid when products are purchased and are used to manage a governmental program. EPR would involve a variable fee set by producers based on the true cost of recovery, with their programs financed and managed by producers. Fees typically fund recycling collection systems and provide no economic incentives for the consumer or for system efficiency. EPR, on the other hand, shifts the focus upstream, providing an incentive to manufacturers to reduce recycling costs and to improve product/packaging design for source reduction and increased recyclability. If applied to TPW, the fee could potentially contribute to butt collection and transfer centers, as well as to the establishment of monitored, hazardous waste storage sites for TPW.

### Fines for Littering

Fines are levied by state and local communities for littering on roadways, beaches, parks, and other public spaces [58]. Fines could also be levied against cigarette manufacturers based on the quantity of brand-specific cigarette waste found on cleanups or as improperly disposed waste from ashtrays, cigarette butt receptacles, or other sources. The fines would at least partially compensate taxpayers for clean-up, collecting, and disposing of cigarette waste. At a national level, the Comprehensive Environmental Resource Compensation and Liability Act of 1980 (Superfund Program), provides a broad framework for requiring companies or other parties to clean-up pollution activities for which they are responsible and/or to pay fines and damages associated with the pollution being cleaned up by others [59].

### Changing social norms

Changing the perceptions about TPW as harmless litter will involve extraordinary social normative changes in the smoking ritual itself [60]. Smokers and non-smokers alike must recognize the externalities of discarding TPW. Cigarette butts are not simply a minor littering problem but rather an externality burdening non-smokers and communities. TPW is the most common waste product (by count) globally. The policy interventions listed above will all contribute to a changing social norm about smoking. Smoking itself has become less and less socially acceptable; TPW disposal into the environment should also become less and less socially acceptable, and its differential impact on poor and minority communities may also classify it as a social justice issue.

## Conclusion

This review suggests that there is precedent for enacting local, state, and national laws, regulations and other mandatory or voluntary interventions to protect the environment from toxic and nonbiodegradable solid TPW through EPR/PS. TPW has not as yet been subject to any systematic take back or safe disposal regulations that create EPR for the tobacco industry; nor has it been subject to PS, whereby others along the supply and retail chain, including distributors, retailers, employers, governments, or other parties may share responsibility to prevent TPW contamination of the environment. Despite EPR and PS taking different approaches for responsibility, the two principles are best viewed as complementary in that they can work in tandem to prevent, reduce, and mitigate TPW’s environmental effects.

The first tenet of EPR calls for internalizing the environmental cost of products into their retail price, and the second tenet calls for shifting the economic burden of managing toxicity and other environmental harm associated with post-consumer waste away from local governments and taxpayers and on to producers. For TPW, they are both very applicable, and very appropriate. Regrettably, the third tenant, providing incentives to producers to incorporate environmental considerations in the design of the product, is unachievable, given the toxic, hazardous chemicals permanently embedded in the tobacco product. Nonetheless, a specific sales ban on single use filters, which are not a health-protective device, may reduce the non-biodegradable portion of TPW. In 2014, a bill was introduced in the California Assembly to ban the sale of single-use cigarette filters for environmental reasons; states have the authority to restrict the sales but not the manufacturing of tobacco products. While the bill did not emerge from committee deliberations, this novel approach is very likely to be considered again in California and other jurisdictions [61].

Based on application of EPR and PS principles to other products, PS is more likely to be the operative system for TPW prevention, reduction, and mitigation, though steps may be taken to place financial responsibilities on to the tobacco industry. While safe cleanup and disposal approaches to TPW would benefit greatly from an EPR and/or PS regime, the tobacco industry is likely to fiercely resist any measures that would shift responsibility directly back to the industry, or to other parties involved in the lifecycle, for the environmental costs or impacts of TPW [62].

The Polluter Pays and Precautionary Principles may apply to TPW. The PPP supports the view that the commercial polluter should bear the environmental and social costs of its activities, with those costs reflected in the market price for goods and services. The PPP, as well as the Precautionary Principle, support EPR/PS application, given the evidence that the TPW waste stream has toxic, carcinogenic, and otherwise harmful chemicals derived from tobacco products and the attached cellulose acetate filters. As such, prevention, reduction and mitigation efforts involving TPW should be undertaken to help prevent potential harm to humans, animals, and ecosystems before it is evident.

For some of ten policy actions we have reviewed for EPR/PW application to TPW, the connection is direct while for others it is indirect. The 1st policy action, calling for laws and programs that mandate EPR and/or PS as well as voluntary actions by the tobacco industry can be compared to policies applied to toxic as well as non-toxic products as suggested by our three case studies. The 5th policy action, litigation, places responsibility via negligence, nuisance, product liability, and other legal theories on to the tobacco industry for failure to take reasonable steps to prevent harm or protect rights to use and enjoy real property (e.g., the beach environment). Evidence of those types of harms may be sufficient to make the industry liable for safe clean-up, take-back and/or disposal of their products under an EPR/PS system. Four of the other ten policy actions focus on litter fees, deposit-return refunds, waste fees and fines, which involve the exchange of money among parties who are stakeholders in the life cycle of tobacco products. In that context, PS applies, as these parties share responsibility for managing the cleanup and disposal of TPW. The remaining four policy actions focus on bans of disposable filters, bans on public smoking, mandates for labeling, and efforts to change social norms. All ten policy options contribute to changing of social norms about TPW and may help frame new channels through which society may achieve the end of the tobacco use epidemic.

This review is limited by the existing disconnect between the perception of TPW as harmless waste and the growing recognition that it is toxic and hazardous. Consumers, environmental policymakers, and even smokers do not fully recognize the environmental issues around TPW, and hence, EPR/PS strategies have not been considered for TPW. The focus of the review is further limited to the post-consumer, downstream, end-of-life management of TPW. As referenced in the Introduction, however, there are also environmental impacts at or near the upstream, front-end of the product life cycle, involving the growing process, manufacturing processes, and product design. Ideally, an integrated, comprehensive system of EPR/PS-related management is needed to prevent, reduce and mitigate TPW throughout its life cycle.

Nevertheless, while strategies to reduce smoking and mitigate TPW may vary significantly in their methods and aspirations, they share two core goals: 1) the status quo is unacceptable, and 2) reducing TPW, its environmental impacts, and smoking overall, will require bold, new, and fundamentally different strategies to assure success. These will require a diverse mix of ideas for achieving the goal of a TPW-free environment and better understanding of the life-cycle environmental hazards of tobacco productions, marketing, and consumption. We have asserted that EPR/PS may provide important pathways to achieve these goals.
